# Guide frames improve centerline estimation of complex postures in WormTracer

**DOI:** 10.17912/micropub.biology.002235

**Published:** 2026-08-31

**Authors:** Chung-Kuan Chen, Yuichi Iino, Yu Toyoshima

**Affiliations:** 1 Department of Biological Sciences, Graduate School of Science, The University of Tokyo, Tokyo, 113-0022, Japan; 2 Department of Computational Biology and Medical Sciences, Graduate School of Frontier Sciences, The University of Tokyo, Chiba, 277-8562, Japan

## Abstract

Precise estimation of body posture is essential for analyzing animal behavior. *C. elegans* exhibits a variety of complex postures, such as turning and looping, for which centerline extraction is challenging. WormTracer, an algorithm designed to ensure temporal continuity of centerlines, enabled reliable centerline extraction. However, accurate estimation remains difficult without human intervention in animals exhibiting prolonged, complex postures. Here, we present an updated version of WormTracer that accepts user-revised centerlines as guide frames, enabling accurate estimation even during prolonged complex postures. In addition, we provide a Napari plugin, Napari-WormTracer, which allows users to manually revise centerlines estimated by WormTracer.

**Figure 1. Improved WormTracer enables accurate estimation of complex posture using a revised guide frame:** (A) Time-series analysis of a representative worm exhibiting prolonged complex postures. The first panel shows posture classification (simple vs. complex), with guide frames indicated in green. The second panel shows image loss (MSE) for centerlines obtained using the canonical thinning algorithm (gray) and after WormTracer optimization either without (blue) or with (orange) guide frames. (B) Time-series raw images and corresponding binarized mask overlaid with the centerlines estimated without or with guide frame at time point 797. Circle indicates head position. Centerlines obtained without guide frames are shown in blue, whereas those obtained with guide frames are shown in orange. A manually annotated centerline used as a guide frame is shown in green. (C) Interface of Napari-Wormtracer, a Napari-based plugin for revising centerlines exported from WormTracer, is shown.

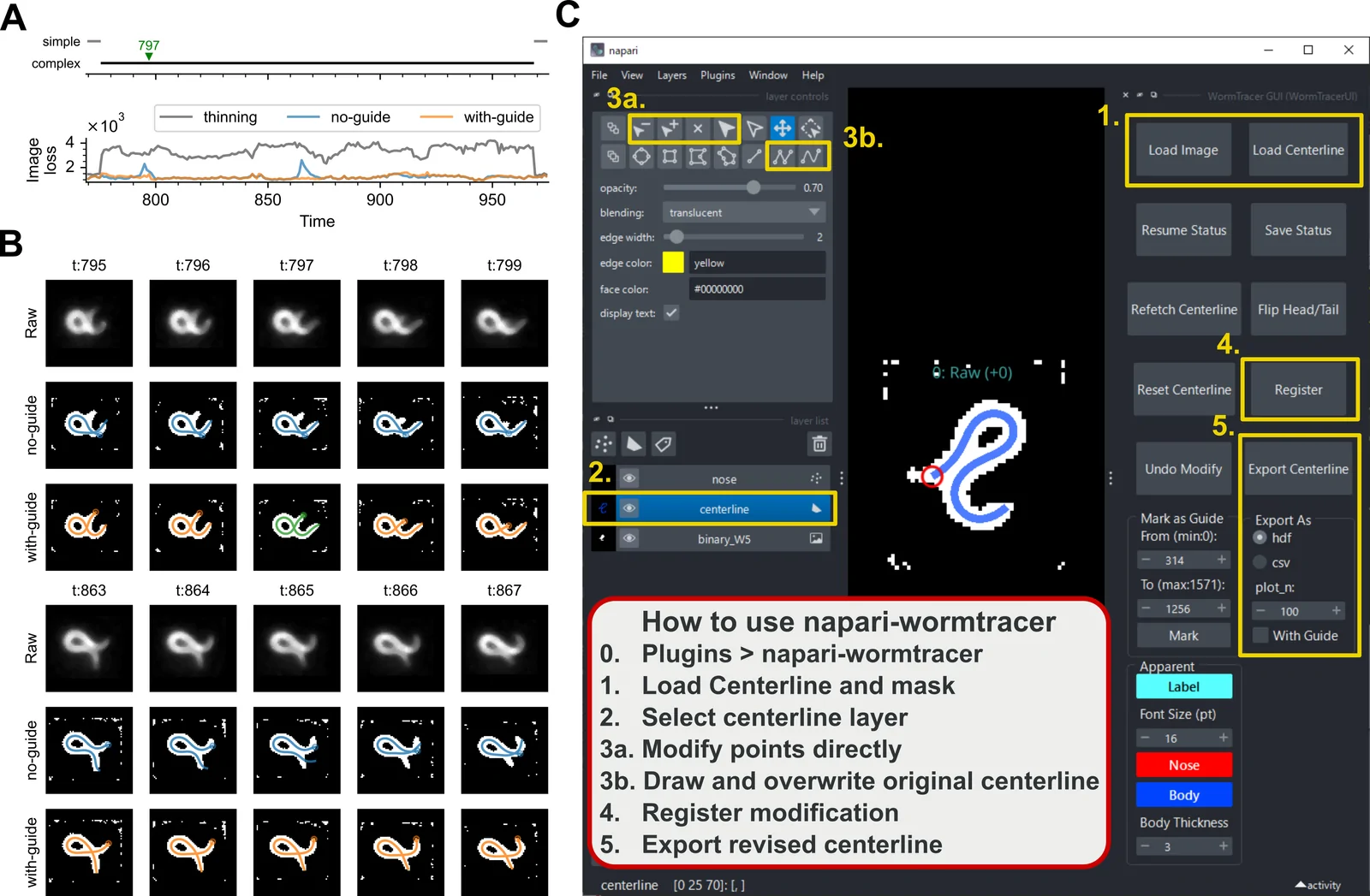



## Description

Behavioral analysis relies on accurate extraction of body posture, which is essential for uncovering the mechanisms underlying motion control and for quantitatively modeling the movement. However, precise posture estimation remains challenging due to the diversity of postures, frequent occlusion of body parts, and variability in imaging conditions (Mathis et al., 2018; Pereira et al., 2022). In *C. elegans* research, many studies have been dedicated to estimating the body posture using variety of computational approaches. For example, Tierpsy provides a well-established pipeline to analyze the worm posture using traditional imaging processing techniques (Javer, Currie, et al., 2018; Javer, Ripoll-Sánchez, et al., 2018). Moreover, deep learning models for instance segmentation have improved posture estimation by increasing the accuracy of binary masks (Castro et al., 2025; Deserno & Bozek, 2023). In addition to the mask-based extraction, several methods directly predict the centerlines from raw input images without prior binarization (Alonso & Kirkegaard, 2023; Hebert et al., 2021; Layana Castro et al., 2023; Saha et al., 2025; Weheliye et al., 2025). However, training such deep learning model typically requires large amounts of manually annotated data or generation of synthetic data from limited annotations. To address this limitation, in our previous study we developed WormTracer (Kuze et al., 2026), an algorithm that estimates the centerline by comparing a worm-shaped mask reconstructed from the candidate centerline with the observed worm silhouette, while enforcing temporal continuity and spatial smoothness of the centerlines. First, WormTracer preprocessed the input masks to extract the centerlines using a canonical thinning algorithm. The extracted centerlines were then classified as either “simple” or “complex”. For example, during turning behavior, contact between the head or tail and the body generated looped masks that could not be accurately resolved using the thinning algorithm alone. Consequently, these frames were classified as “complex”. To objectively identify the complex frames based on the estimated centerlines, WormTracer evaluated the pixel mismatch between the original binary body mask and the mask reconstructed from the centerlines. Frames in which the thinning algorithm failed to accurately reconstruct the original mask were classified as complex ( Fig. 1A ). Consecutive frames with the same classification were then grouped into behavioral sequences. Following this preprocessing step, initial centerlines for the simple sequences were derived directly from the thinning method, whereas those for the complex sequences were generated by linearly interpolating centerlines from adjacent simple sequences. These estimates were then optimized via gradient descent to minimize mask pixel mismatch, with a regularization penalty for abrupt spatial and temporal changes. Eventually, the output centerlines were optimized to best fit the input mask while maintaining their spatial and temporal continuity. The entire optimization required only a time-series binary mask, and the centerlines were automatically estimated without human intervention.

Despite these advantages, WormTracer has several limitations. When complex postures persist for a long period, relying on adjacent simple frames for initial estimates becomes insufficient. The errors are more likely to occur in highly coiled shapes, where mask-based reconstruction alone may fail to resolve the correct centerline topology. An example dataset shown here exhibited prolonged complex posture from time points 776 to 968 and was analyzed using WormTracer to estimate centerlines ( Fig. 1A, blue). In this case, frames around time point 797 showed increased image loss. Consistently, these frames also displayed incorrect centerline predictions ( Fig. 1B, blue).

To address these limitations, we updated WormTracer to incorporate manually specified centerlines (termed guide frames) for optimization. The guide frames serve as anchoring points that are treated as ground truth during optimization and partitions the full sequence into smaller optimization blocks. During optimization, these guide frames correct the topology of the centerlines, allowing the continuity constraints of the WormTracer algorithm to propagate the correct head-tail orientation and bending angles to neighboring frames. As a result, providing a single guide frame at time point 797 ( Fig. 1A and B, green) corrected the centerline topology not only at that frame but also during the complex postures around time point 865 within the same behavioral sequence ( Fig. 1A and B, orange). This result indicates that guide frames can resolve erroneous centerline topology and allow WormTracer to automatically propagate the correction throughout the sequence, substantially reducing the amount of manual intervention required.

In conclusion, incorporating guide frames improves overall prediction accuracy and enables WormTracer to recover correct centerlines with minimal manual intervention. The updated version of WormTracer is available at: https://github.com/lycantrope/wormtracer/releases/tag/v21.0.0

In addition to enabling WormTracer to incorporate guide frames for centerline refinement, we developed a Napari plugin (Sofroniew et al., 2026), Napari-WormTracer ( Fig. 1C ; https://github.com/lycantrope/napari-wormtracer ). This tool enables users to easily inspect centerlines generated by WormTracer. It also provides functionality to manually revise centerlines and register these corrections, facilitating the generation of guide frame data for subsequent WormTracer optimization. Furthermore, it can serve as a practical tool to prepare training datasets for other established methods.

In summary, guide frames enhance the robustness of WormTracer for complex postures, and Napari-WormTracer provides a practical interface for manual correction, enabling accurate centerline estimation with minimal user intervention.

## Methods

**Strains and culture**

All *C. elegans* strains were mainly cultured on nematode growth medium (NGM) with *Escherichia coli* strain OP50 , as described (Brenner, 1974) and maintained at 20°C. For sample movies, bright field images were acquired from adult individuals of TOY21, a strain derived from QD15#4 (Toyoshima et al., 2020; Yang et al., 2026) and was developed for whole-brain calcium imaging.

**Definition of loss functions for centerline estimation**

WormTracer defines five loss functions to evaluate the fitness of estimated centerlines to the observed worm mask, which ensures both spatial accuracy and temporal continuity (Kuze et al., 2026). These include image loss, temporal angular continuity loss (continuity loss), spatial angular continuity loss (smoothing loss), length continuity loss (length loss), and centroid loss (center loss). Specifically, image loss is calculated as the mean squared error (MSE) between two masks, the observed worm silhouette and the mask reconstructed from the estimated centerline. Temporal and spatial angular continuity losses are defined as the MSE differences of segment bending angles along the temporal dimension (i.e., between consecutive time points) and the spatial dimension (i.e., along the centerline within a single frame), respectively, constraining abrupt changes in angle. Length continuity loss and centroid loss enforce consistency in segment length and stability of the center of mass by minimizing their variance and displacement, respectively.

**Implementation of WormTracer with guide frames**

The major difference from the previous version of WormTracer was the support for incorporating guide frames. Particularly, the newer of WormTracer loaded the correct centerlines from external files. After calculating the image loss as, *Im _loss,t_ = MSE(mask _model,t_ − mask _real,t_ )* , the losses in the frames with correct centerlines (guide frames) were replaced by the minimum loss, expressed as *Im _loss,t_g_ = min(Im _loss,t_ ), t_g ∈ guide_frames* . Subsequently, the long behavioral sequences were split into small optimization blocks at these guide frames, thereby anchoring the topology of neighboring centerlines, such as head-tail orientation, segment length and bending angles.

WormTracer was implemented in Python using several libraries for data analysis and image processing, including NumPy (Harris et al., 2020), SciPy (Virtanen et al., 2020), scikit-image (Van Der Walt et al., 2014), and OpenCV (Bradski, 2000). Deep learning components were implemented using PyTorch (Paszke et al., 2019). Visualization was performed using Matplotlib (Hunter, 2007), and the final figure layouts were assembled using Inkscape ( https://inkscape.org/ ). All code and detailed usage instructions are available at https://github.com/lycantrope/wormtracer and https://github.com/lycantrope/napari-wormtracer . In this paper, we used WormTracer v21.0.0 and napari-wormtracer 2026.05.29.

## Reagents

**Table:** 

**Strain**	**Genotype**	**Source**
OP50	*Escherichia coli*	CGC
TOY21	* qjIs11[glr-1p::svnls2::TagBFPsyn, ser-2 (prom2)p::svnls2::TagBFPsyn]* V; *peIs3042[eat-4p::svnls2::TagRFP675syn,lin-44p::GFP* ] X.; *Is[H20p::nls3::tdTomato,H20p::nls2::GCaMP6f]#72-2-5*	(Yang et al., 2026)

## Data Availability

Description: A python package for centerlines estimation. Resource Type: Software. DOI: https://doi.org/10.22002/11rsh-36g19 Description: A napari-plugin for centerlines refinement. Resource Type: Software. DOI: https://doi.org/10.22002/f4mjs-j0x86

## References

[R1] Alonso Albert, Kirkegaard Julius B. (2023). Fast detection of slender bodies in high density microscopy data. Communications Biology.

[R2] Brenner S (1974). THE GENETICS OF
*CAENORHABDITIS ELEGANS*. Genetics.

[R3] Castro Pablo E. Layana, Kounakis Konstantinos, Garví Antonio García, Gkikas Ilias, Tsiamantas Ioannis, Tavernarakis Nektarios, Sánchez-Salmerón Antonio-José (2025). SegElegans: Instance segmentation using dual convolutional recurrent neural network decoder in Caenorhabditis elegans microscopic images. Computers in Biology and Medicine.

[R4] Deserno Maurice, Bozek Katarzyna (2023). WormSwin: Instance segmentation of C. elegans using vision transformer. Scientific Reports.

[R5] Harris Charles R., Millman K. Jarrod, van der Walt Stéfan J., Gommers Ralf, Virtanen Pauli, Cournapeau David, Wieser Eric, Taylor Julian, Berg Sebastian, Smith Nathaniel J., Kern Robert, Picus Matti, Hoyer Stephan, van Kerkwijk Marten H., Brett Matthew, Haldane Allan, del Río Jaime Fernández, Wiebe Mark, Peterson Pearu, Gérard-Marchant Pierre, Sheppard Kevin, Reddy Tyler, Weckesser Warren, Abbasi Hameer, Gohlke Christoph, Oliphant Travis E. (2020). Array programming with NumPy. Nature.

[R6] Hebert Laetitia, Ahamed Tosif, Costa Antonio C., O’Shaughnessy Liam, Stephens Greg J. (2021). WormPose: Image synthesis and convolutional networks for pose estimation in C. elegans. PLOS Computational Biology.

[R7] Hunter John D. (2007). Matplotlib: A 2D Graphics Environment. Computing in Science & Engineering.

[R8] Javer Avelino, Currie Michael, Lee Chee Wai, Hokanson Jim, Li Kezhi, Martineau Céline N., Yemini Eviatar, Grundy Laura J., Li Chris, Ch’ng QueeLim, Schafer William R., Nollen Ellen A. A., Kerr Rex, Brown André E. X. (2018). An open-source platform for analyzing and sharing worm-behavior data. Nature Methods.

[R9] Javer Avelino, Ripoll-Sánchez Lidia, Brown André E.X. (2018). Powerful and interpretable behavioural features for quantitative phenotyping of
*Caenorhabditis elegans*. Philosophical Transactions of the Royal Society B: Biological Sciences.

[R10] Kuze Koyo, Tazawa Ukyo T., Suwazono Karin, Chen Chung-Kuan, Toyoshima Yu, Iino Yuichi (2026). WormTracer: A precise method for worm posture analysis using temporal continuity. Journal of Neuroscience Methods.

[R11] Layana Castro Pablo E., García Garví Antonio, Navarro Moya Francisco, Sánchez-Salmerón Antonio-José (2023). Skeletonizing Caenorhabditis elegans Based on U-Net Architectures Trained with a Multi-worm Low-Resolution Synthetic Dataset. International Journal of Computer Vision.

[R12] Mathis Alexander, Mamidanna Pranav, Cury Kevin M., Abe Taiga, Murthy Venkatesh N., Mathis Mackenzie Weygandt, Bethge Matthias (2018). DeepLabCut: markerless pose estimation of user-defined body parts with deep learning. Nature Neuroscience.

[R13] Paszke Adam, Gross Sam, Massa Francisco, Lerer Adam, Bradbury James, Chanan Gregory, Killeen Trevor, Lin Zeming, Gimelshein Natalia, Antiga Luca, Desmaison Alban, Köpf Andreas, Yang Edward, DeVito Zach, Raison Martin, Tejani Alykhan, Chilamkurthy Sasank, Steiner Benoit, Fang Lu, Bai Junjie, Chintala Soumith (2019). PyTorch: An Imperative Style, High-Performance Deep Learning Library.

[R14] Pereira Talmo D., Tabris Nathaniel, Matsliah Arie, Turner David M., Li Junyu, Ravindranath Shruthi, Papadoyannis Eleni S., Normand Edna, Deutsch David S., Wang Z. Yan, McKenzie-Smith Grace C., Mitelut Catalin C., Castro Marielisa Diez, D’Uva John, Kislin Mikhail, Sanes Dan H., Kocher Sarah D., Wang Samuel S.-H., Falkner Annegret L., Shaevitz Joshua W., Murthy Mala (2022). SLEAP: A deep learning system for multi-animal pose tracking. Nature Methods.

[R15] Saha Debasish, Chaudhary Shivam, Vyas Dhyey, Ghosh-Roy Anindya, Sharma Rati (2025). Deep-Pose-Tracker: an automated behavioural analysis framework for
*Caenorhabditis elegans*.

[R16] Sofroniew Nicholas, Lambert Talley, Bokota Grzegorz, Nunez-Iglesias Juan, Sobolewski Peter, Sweet Andrew, Gaifas Lorenzo, Evans Kira, Burt Alister, Doncila Pop Draga, Yamauchi Kevin, Weber Mendonça Melissa, Rodríguez-Guerra Jaime, Liu Lucy, Buckley Genevieve, Vierdag Wouter-Michiel, Anderson Ashley, Monko Timothy, Willing Carol, Rodríguez-Reza Carlos M., Royer Loic, Can Solak Ahmet, Harrington Kyle I. S., Abramo Jacopo, Ahlers Jannis, Ajina Sesan, Althviz Moré Daniel, Ambroset Mélodie, Amsalem Oren, Andò Edward, Annex Andrew, Archit Anwai, Aronssohn Constantin, Balzaretti Filippo, Boone Peter, Bestak Kresimir, Bragantini Jordão, Bunten David, Bussonnier Matthias, Caporal Clément, Chazotte Margot, Coccimiglio Ian, Čočková Zuzana, Defauw Arne, Czasak Sara, Eglinger Jan, Eisenbarth Andreas, Freeman Jeremy, Fukai T. Yohsuke, Garnett Arbor, Gohlke Christoph, Gunalan Kabilar, Halchenko Yaroslav Olegovich, Har-Gil Hagai, Harfouche Mark, Hilsenstein Volker, Huijben Teun A.P.M., Hutchings Katherine, Kawai Hiroki, Kozar Robert, Lauer Jessy, Le Meur-Diebolt Samuel, Lefebvre Austin E. Y. T., Lichtner Gregor, Liu Hanjin, Liu Ziyang, Lowe Alan, Malin-Mayor Caroline, Marconato Luca, Martin Sean, McGovern Abigail, Migas Lukasz, Miller Nadalyn, Miñano Sofía, Muñoz Hector, Müller Jan-Hendrik, Nauroth-Kreß Christopher, Newstein Peter, Obenhaus Horst A., Palecek David, Pape Constantin, Pellegrini Yann, Perlman Eric, Theart Rensu Petrus, Pevey Kim, Peña-Castellanos Gonzalo, Phelps Jasper, Pierré Andrea, Pinto David, Reksoatmodjo Anthony, Ross David, Russell Craig T., Ryan James, Saveur Tom, Schabel Matthias Christian, Selzer Gabriel, Sharma Rupesh, Sheikh Imad Uddin, Sirmpilatze Nikoloz, Smith MB, Smith Paul, Sofiiuk Konstantin, Soltwedel Johannes, Stansby David, Steuer Michal, Teo Wulin, Vanaret Jules, Wadhwa Pam, Weigert Martin, Windhager Jonas, Winston Philip, Yu Qin, Zhang Liudeng, Zhao Rubin, Witz Guillaume, Leomil Zoccoler Marcelo, Yadav Aniket Singh, Hinderling Lucien, Kabde Omkar, Roald-Arbøl Mikkel, Rochat Giliane, Castelli Filippo Maria, Venugopal Revathy, Coussoux Edouard, Seth Mridul, Becht Job, Creeten Christophe, Morais Sébastien, Bloemsaat Bas, Kania Kamil, Liaskas Ioannis, Hada Aroj, Nagineni Venkateswarlu (2026). napari: a multi-dimensional image viewer for Python.

[R17] Toyoshima Yu, Wu Stephen, Kanamori Manami, Sato Hirofumi, Jang Moon Sun, Oe Suzu, Murakami Yuko, Teramoto Takayuki, Park Chanhyun, Iwasaki Yuishi, Ishihara Takeshi, Yoshida Ryo, Iino Yuichi (2020). Neuron ID dataset facilitates neuronal annotation for whole-brain activity imaging of C. elegans. BMC Biology.

[R18] van der Walt Stéfan, Schönberger Johannes L., Nunez-Iglesias Juan, Boulogne François, Warner Joshua D., Yager Neil, Gouillart Emmanuelle, Yu Tony (2014). scikit-image: image processing in Python. PeerJ.

[R19] Virtanen Pauli, Gommers Ralf, Oliphant Travis E., Haberland Matt, Reddy Tyler, Cournapeau David, Burovski Evgeni, Peterson Pearu, Weckesser Warren, Bright Jonathan, van der Walt Stéfan J., Brett Matthew, Wilson Joshua, Millman K. Jarrod, Mayorov Nikolay, Nelson Andrew R. J., Jones Eric, Kern Robert, Larson Eric, Carey C J, Polat İlhan, Feng Yu, Moore Eric W., VanderPlas Jake, Laxalde Denis, Perktold Josef, Cimrman Robert, Henriksen Ian, Quintero E. A., Harris Charles R., Archibald Anne M., Ribeiro Antônio H., Pedregosa Fabian, van Mulbregt Paul, Vijaykumar Aditya, Bardelli Alessandro Pietro, Rothberg Alex, Hilboll Andreas, Kloeckner Andreas, Scopatz Anthony, Lee Antony, Rokem Ariel, Woods C. Nathan, Fulton Chad, Masson Charles, Häggström Christian, Fitzgerald Clark, Nicholson David A., Hagen David R., Pasechnik Dmitrii V., Olivetti Emanuele, Martin Eric, Wieser Eric, Silva Fabrice, Lenders Felix, Wilhelm Florian, Young G., Price Gavin A., Ingold Gert-Ludwig, Allen Gregory E., Lee Gregory R., Audren Hervé, Probst Irvin, Dietrich Jörg P., Silterra Jacob, Webber James T, Slavič Janko, Nothman Joel, Buchner Johannes, Kulick Johannes, Schönberger Johannes L., de Miranda Cardoso José Vinícius, Reimer Joscha, Harrington Joseph, Rodríguez Juan Luis Cano, Nunez-Iglesias Juan, Kuczynski Justin, Tritz Kevin, Thoma Martin, Newville Matthew, Kümmerer Matthias, Bolingbroke Maximilian, Tartre Michael, Pak Mikhail, Smith Nathaniel J., Nowaczyk Nikolai, Shebanov Nikolay, Pavlyk Oleksandr, Brodtkorb Per A., Lee Perry, McGibbon Robert T., Feldbauer Roman, Lewis Sam, Tygier Sam, Sievert Scott, Vigna Sebastiano, Peterson Stefan, More Surhud, Pudlik Tadeusz, Oshima Takuya, Pingel Thomas J., Robitaille Thomas P., Spura Thomas, Jones Thouis R., Cera Tim, Leslie Tim, Zito Tiziano, Krauss Tom, Upadhyay Utkarsh, Halchenko Yaroslav O., Vázquez-Baeza Yoshiki, SciPy 1.0 Contributors (2020). SciPy 1.0: fundamental algorithms for scientific computing in Python. Nature Methods.

[R20] Weheliye Weheliye H., Rodriguez Javier, Feriani Luigi, Javer Avelino, Uhlmann Virginie, Brown André E. X. (2025). A neural network model enables worm tracking in challenging conditions and increases signal-to-noise ratio in phenotypic screens. PLOS Computational Biology.

[R21] Yang Xiaoxiong, Zeng Jingxuan, Chen Chung‐Kuan, Iino Yuichi, Toyoshima Yu (2026). Developing a Robust Multiround HCR‐FISH Method Modified for
*Caenorhabditis elegans*. Genes to Cells.

